# Supplementary material for: Comparison of complex networks and tree-based methods of phylogenetic analysis and proposal of a bootstrap method
Source: PeerJ. 2018 Feb 9;6:e4349. doi: 10.7717/peerj.4349 (PMC5808311; doi:10.7717/peerj.4349)
Supplement: Figure S1 [file peerj-06-4349-s002.pdf]

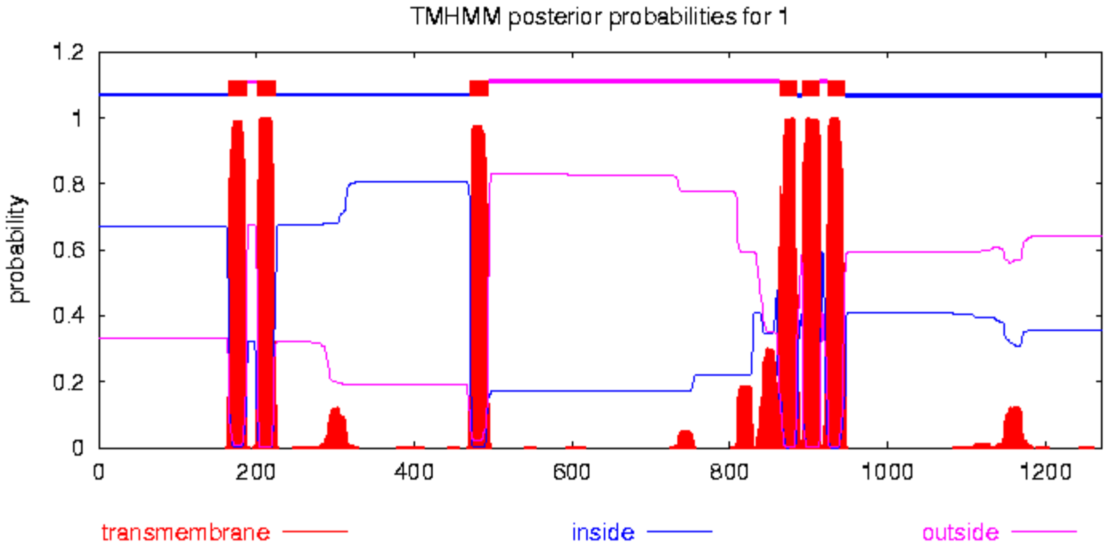

SF1 a: Profile 1 of transmembrane topological organization of complete sequences of protein of CHSBasidio database.

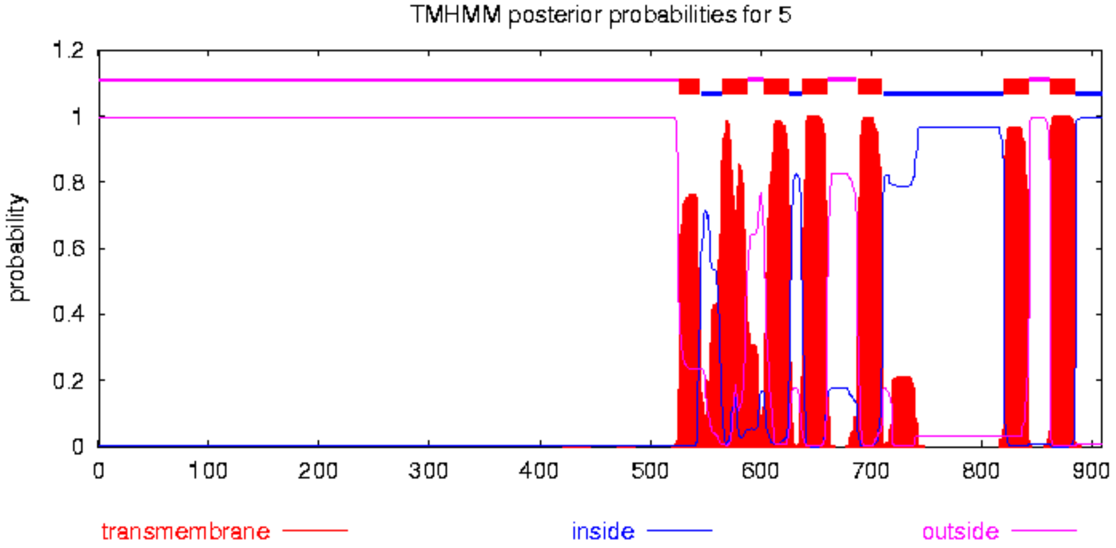

SF1 b: Profile 2 of transmembrane topological organization of complete sequences of protein of CHSBasidio database.

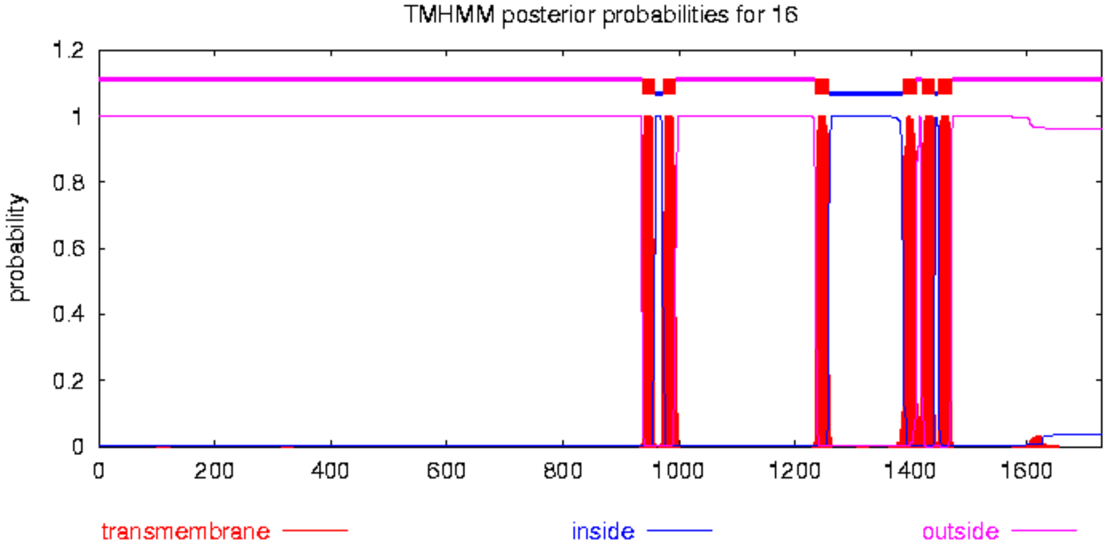

SF1 c: Profile 3 of transmembrane topological organization of complete sequences of protein of CHSBasidio database.

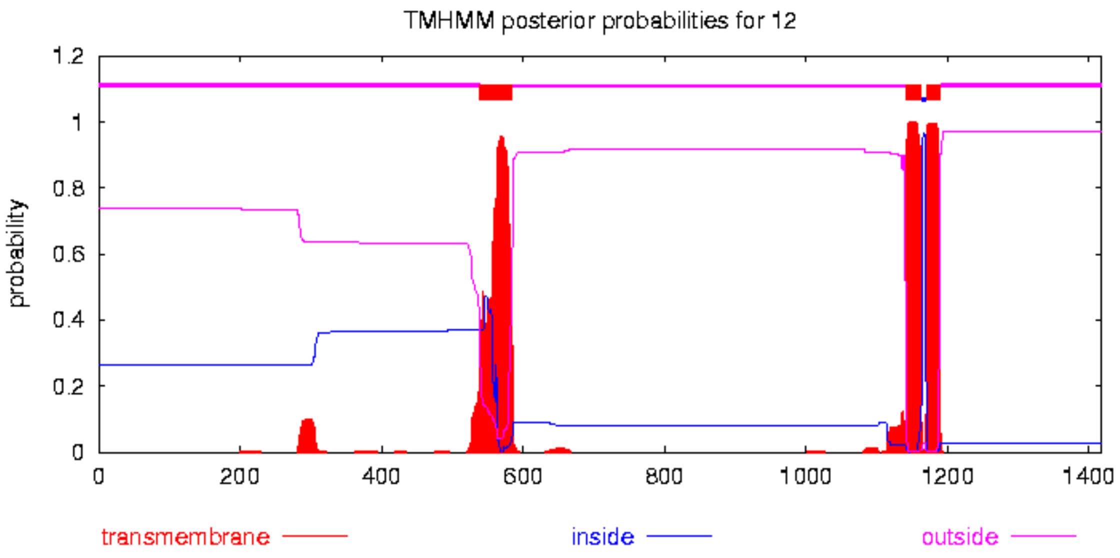

SF1 d: Profile 4 of transmembrane topological organization of complete sequences of protein of CHSBasidio database.

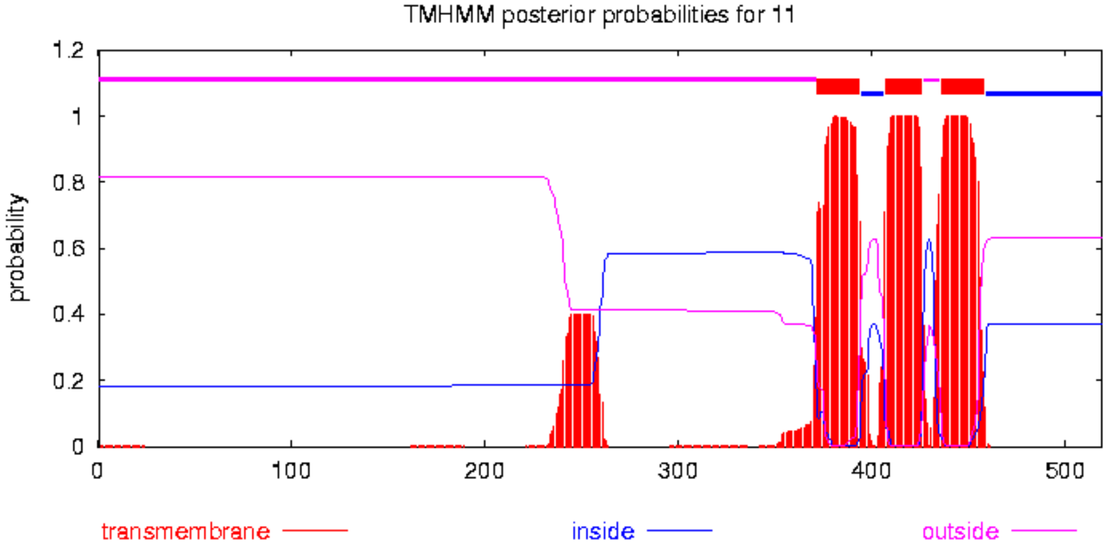

SF1 e: Profile 5 of transmembrane topological organization of complete sequences of protein of CHSBasidio database.

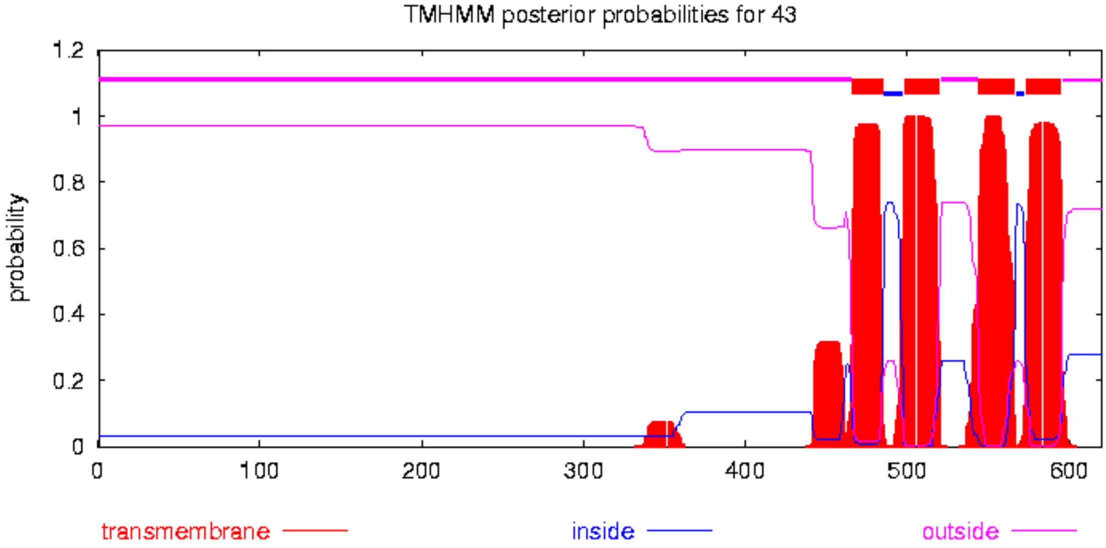

SF1 f: Profile 6 of transmembrane topological organization of complete sequences of protein of CHSBasidio database.

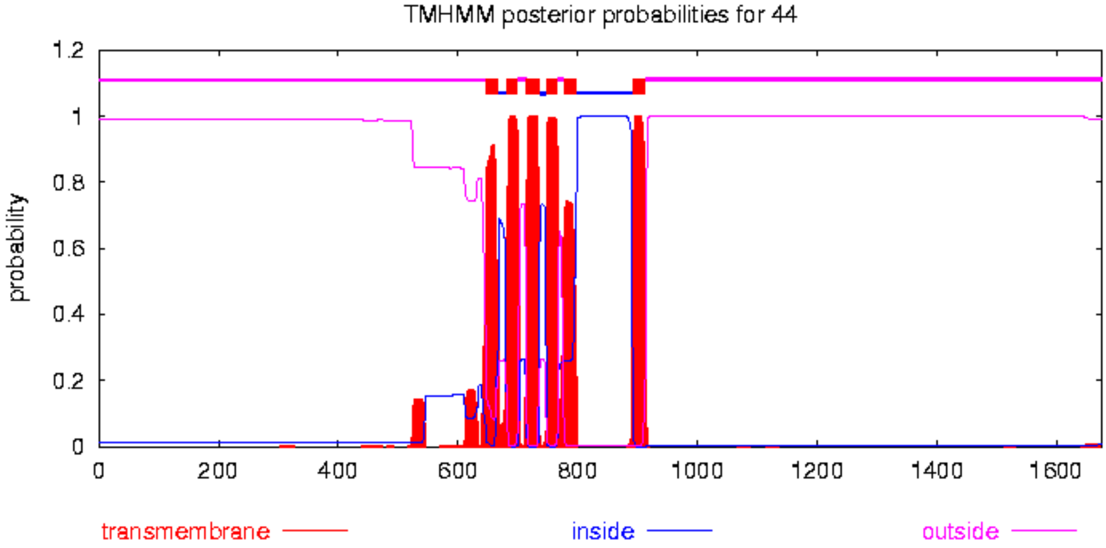

SF1 g: Profile 7 of transmembrane topological organization of complete sequences of protein of CHSBasidio database.
